# Supplementary material for: Molecular basis of ion permeability in a voltage‐gated sodium channel
Source: EMBO J. 2016 Feb 12;35(8):820–30. doi: 10.15252/embj.201593285 (PMC4972137; doi:10.15252/embj.201593285)
Supplement: Supplementary file 1 — Appendix [file EMBJ-35-820-s001.pdf]

Appendix
for
Molecular Basis of Ion Permeability in a Voltage-Gated Sodium Channel

Claire E. Naylor[1+], Claire Bagnéris[1+], Paul G. DeCaen[2], Altin Sula[1], Antonella Scaglione [2,~], David E. Clapham[2], B. A. Wallace[1*]

+These authors contributed equally
[1]Institute of Structural and Molecular Biology, Birkbeck College, University of London, London, WC1E 7HX, U.K.
[2]Howard Hughes Medical Institute, Department of Cardiology, Boston Children's Hospital, Boston, Massachusetts 02115, USA; Department of Neurobiology, Harvard Medical School, Boston, Massachusetts 02115, USA
[~]Current address: [3]Institute of Molecular Biology and Pathology of CNR, Dept. of Biochemical Sciences, Sapienza University of Rome, 00185 Rome, Italy
*Corresponding author email: b.wallace@mail.cryst.bbk.ac.uk

Appendix Table of Contents
Additional (Supporting Data)
Appendix References
Appendix Figures
Appendix Table

Additional (Supporting) Data:

Crystals of NavMs Prepared in the Presence of Other (Anomalous Scattering) Cations:

To examine the potential binding of other monovalent cations in the SF (or elsewhere in the structure), crystals were grown or soaked in the presence of thallium ions. Thallium ions can sometimes act as substitutes for sodium ions (although their ionic radii are considerably larger) and when bound, will give rise to anomalous scattering signals, providing unambiguous identification of their nature and location. In the Tl^+ co-crystals produced, a number of small positive peaks (low occupancy) in the difference anomalous maps were visible in the vestibule at the entrance to the pore (data not shown), however, no anomalous difference peaks were present in the SFs of the pores. Electron density peaks arising from non-anomalous scatterers were present in the SF at essentially identical positions as those in the sodium-only crystals, suggesting that they were tightly bound sodium ions carried through the purification steps that were not displaced by the thallium ions either during soaking or co-crystallisation (but note that the co-crystallisations also contained sodium ions as crystals would not form in their absence). It is not, perhaps, surprising, that Tl^+ was unable to displace sodium ions in the SF because other sodium channels have been shown to have much lower permeabilities for thallium ions than they do for sodium ions (Hille, 1972). Crystals soaked or grown in the presence of monovalent cations Ag^+ and Rb^+, and in the divalent cations Ba^{2+}, Cd^{2+}, and Mn^{2+} (all of which would have also produced anomalous signals if bound) also exhibited no anomalous difference densities indicating they did not enter the SF, nor did they bind at the vestibule to the pore. Like the thallium ions, they also did not prevent entry of the sodium ions into the SF.

Cd^{2+} and Ag^+ ions are effective antagonists of mammalian heart, neuronal, and skeletal muscle sodium channels ($IC_{50} \approx 100$ µM to 2 mM) (Backx et al, 1992; Yamagishi et al., 2001), and Cd^{2+} has been shown to block NaChBac and Na_VRh-chimera bacterial sodium channels ($IC_{50} \approx 200 – 800$ µM) (Ren et al., 2001; Zhang et al, 2012), but electrophysiology

measurements in this study (Appendix Figure S10) show neither Cd^{2+} nor Ag^{+} block the NavMs channel. Thus, the NavMs pore appears to lack the high-affinity transition metal binding site found in some mammalian and prokaryotic sodium channels, a result that is consistent with the lack of Cd^{2+} or Ag^{+} peaks at the vestibule to the channel.

Identifying the Electron Density within the SF as Arising from Sodium Ions:

Distinguishing between sodium ions which have 10 electrons and water molecules (which also have 10 electrons) can be a challenge crystallographically. At the higher resolution of the crystals in this study (as compared to previous studies which exhibited electron density in the SF, but not of a defined nature), discrete spherical density can be seen at a high sigma cutoff (Appendix Figure S3). This would be more typical of sodium ions than water molecules, which tend to produce more elongated densities.

Additionally, we attempted to refine the contents of the SF in many ways, based on the coordination chemistry of sodium ions. In our final refinement, they were refined as 3 fully occupied sodium ions (cyan balls in Appendix Figure S2A), with partially occupied water molecules placed at geometrically-appropriate positions where there was additional density in both the $2F_o$-F_c and difference maps, with all geometrical restraints for both water and sodium ions turned off to enable to enable them to optimise the interatomic approach distances (see paragraph below). The B-factors of the sodiums refined to ~50 (Table S1 and Figure S6). Examples of other strategies tried included refining as in Appendix Figure S2A but with antibumping functionality in the SF turned on during refinement (Appendix Figure S2B); while optimizing our restraint choice waters were added or removed from the model to improve agreement with the map. Alternately, the structure was refined with 3 sodium ions (with B-factors similar to that of the protein), each with a full complement of cubically-coordinated water molecules (at a distance of ~2.43 A) at half-occupancy (Appendix Figure S2C). Next a refinement was undertaken initially with 3 water molecules only (no sodiums) at the appropriate occupancy and then more waters were placed in the additional regions of density with antibumping on (Appendix Figure S2D). Finally, the structure was refined with 3 sodium ions and water molecules placed at all chemically-suitable positions identified by the programme Hydrosite (Dr. Hongyao Zhu, Pfizer Neusentis, personal communication) (Appendix Figure S2E). Except for the all-water refinement depicted in Appendix Figure S2D which had a significantly higher Rwork/Rfree, the Rwork/Rfree values (listed in the legend to Appendix Figure S2) for all of the other refinements were very similar, as would be expected for a global indicator when small changes have been made to the model. However, the structure with three sodium ions and the waters not constrained by the antibumping function (Appendix Figure S2A), gave the best overall fit to the observed density and was the structure deposited in the PDB, and included in Appendix Table S1. Figure S2A can be compared with the initial difference electron density (no sodium or ions included), contoured at 2.2 σ (Appendix Figure S2F), showing that the locations of the waters in Figure S2A correspond to features present in the initial map, albeit detectable at a somewhat lower density cutoff (compare to the higher cutoff in Figure 2A) than the sodium ions.

Another possible way of distinguishing water and sodium ions is based on the geometry/nature of neighbouring atoms. In this case, because the SF is not tightly packed with density, both water and sodium ions were possible constituents; to test which density was associated with each, anti-bumping features were not included in the refinement seen in Appendix Figure S2A. The most notable feature was that for this refinement the distances between the densities of the "sodium ion" and "water" positions were significantly less than 2.75 Å (the water-water distance) and were in many cases 2.3-2.4 Å, compatible with the sodium ion-water ideal distance, a strong indication that the central densities arose from sodium ions and not water molecules.

The final arguments for the identified sites being due to sodium ions are their similarities with the calcium ion binding sites identified in the CavAb channel (Appendix Figure S9A) and the observation that in the E178D mutant exposure of a positive charge near the top binding site (Figure 3B) removes the density, which would be unlikely if the site was occupied by a water molecule, but would be expected if it was occupied by a sodium ion.

Appendix References:

Backx PH, Yue DT, Lawrence JH, Marban E, Tomaselli GF (1992) Molecular localization of an ion-binding site within the pore of mammalian sodium channels. Science 257: 248-251.

Hille B (1972) The permeability of the sodium channel to metal cations in myelinated nerve. J Gen Physiol 59: 637-658.

Ren D, Navarro B, Xu H, Yue L, Shi Q, Clapham DE (2001) A prokaryotic voltage-gated sodium channel. Science 294: 2372-2375.

Yamagishi T, Li RA, Hsu K, Marban E, Tomaselli GF (2001) Molecular architecture of the voltage-dependent Na channel: functional evidence for alpha helices in the pore. J Gen Physiol 118: 171–182.

Appendix Figures:

Appendix Figure S1: The NavMs channel is sodium selective.

A) Diagram depicting the internal and external conditions for the measurements (see Methods); the concentrations are listed in mM and the corresponding cations (XCl) are matched.

B) *Left*: To confirm the expression of NavMs, extracellular Na^+ solution was exchanged where inward currents were measured by 300 ms depolarizations to -30 mV from a -180 mV holding potential. Of the cations tested (Na^+, Li^+, K^+, Rb^+, Ca^{2+}, Ba^{2+} and Mg^{2+}) only Na^+ and Li^+ generated inward voltage-dependent currents. *Right*: Inward current magnitudes plotted as a function of time as Na^+-containing solution is exchanged for solutions with the indicated ions (colored boxes).

C) *Top*: After NavMs expression was verified from the patched cells (as shown in B), currents measured by positive voltage steps from -180 mV for the indicated extracellular cations. *Bottom*: Resulting current-voltage profiles (expanded below the I-V) showing the reversal potential for each cation (Error ± SEM; n = 5-9).

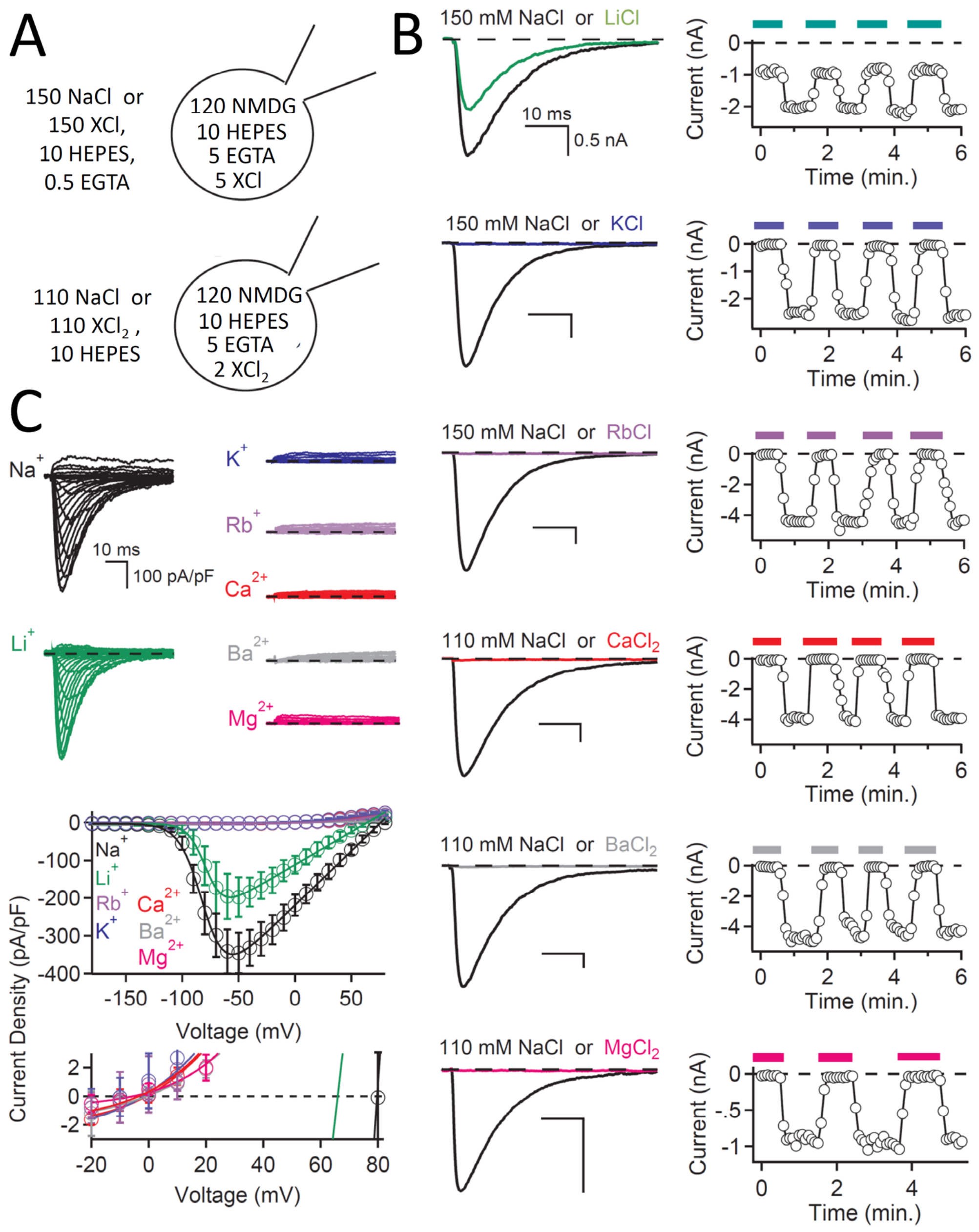

Appendix Figure S2: 2Fo-Fc electron density maps produced by different types of refinements, all shown for the AB tetramer at 1.0 σ.

Sodium ions are shown as cyan spheres and well-ordered water as red spheres (at 0.4x their actual radii, for clarity). Values in square brackets are Rwork/Rfree (%)

A) Sodium ions and waters placed into density, with antibumping off in SF. [17.2/21.2].

B) As in A) but with antibumping on. [17.2/21.0].

C) Sodium ions at full occupancy and ½ occupancy waters placed at cubical coordination sites. [17.3/21.2].

D) 3 water molecules only (no sodiums) at half occupancy with additional waters placed into density. [20.6/22.5].

E) Sodium ions and water molecules placed at all chemically-suitable positions identified by the programme Hydrosite (Dr. Hongyao Zhu (Pfizer Neusentis, personal communication). [17.4/20.6].

F) Initial difference electron density (no sodium or ions included), contoured at 2.2 σ, showing that the locations of the waters in part A above) correspond to features present in the initial map, albeit detectable at a somewhat lower density cutoff (compared to the higher cutoff in Figures 2A and S7) than the sodium ions.

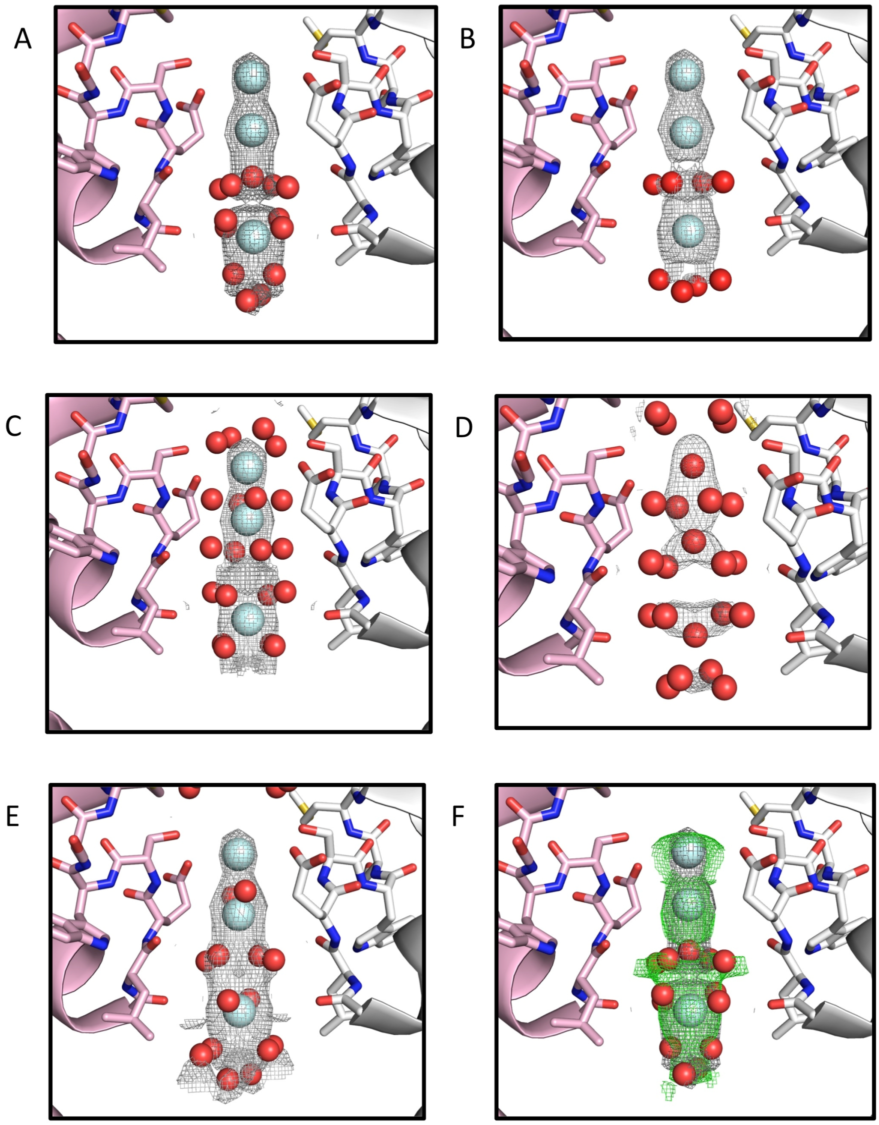

Appendix Figure S3: Electron density in the SF.
2Fo-Fc map at 3.5 sigma (grey mesh) for the refined coordinates (including 3 sodium ions [not shown for clarity] but no water molecules). These views at high sigma depict the spherical nature of the density, although the view on the left gives a slightly distorted appearance due to the crystal symmetry.

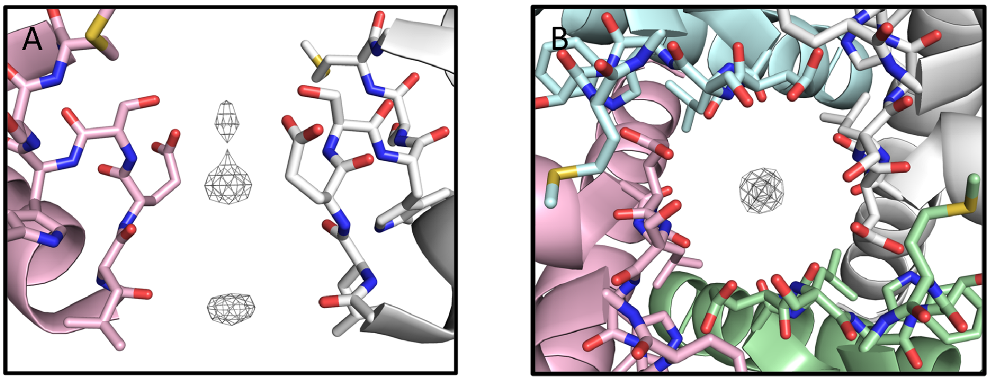

Appendix Figure S4: Final refined electron density map for SF residues and contents.
The 2Fo-Fc electron density map is contoured at 2.0 (dark blue), 1.5 (pink) and 1.0 (green) RMSD, with the final refined model drawn as described in Figure 2.

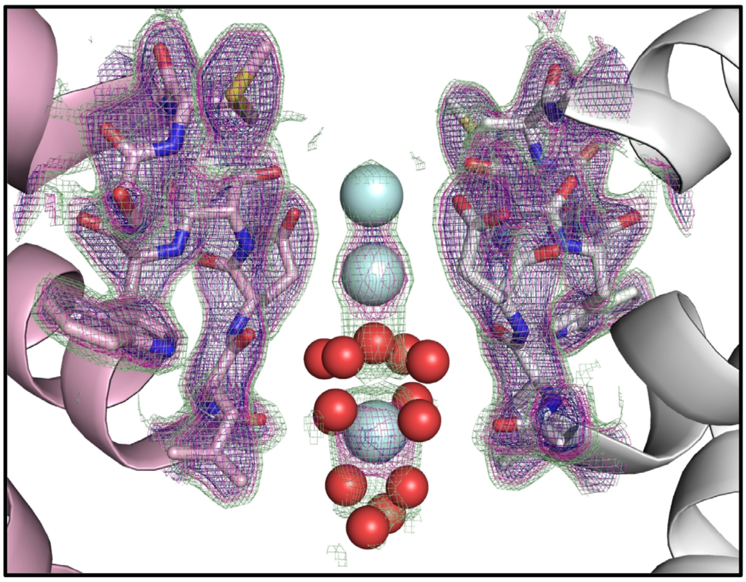

Appendix Figure S5: Omit maps showing the initial densities present in the SF. Maps were calculated after sodium ions and waters were removed from the SF and a refinement round in Buster carried out. 2Fo-Fc maps at 1.5 σ (dark grey mesh) and 1.0 σ (light grey mesh), with the difference map in green mesh. The positions for the sodium ions in the final refined structure are shown as cyan balls.

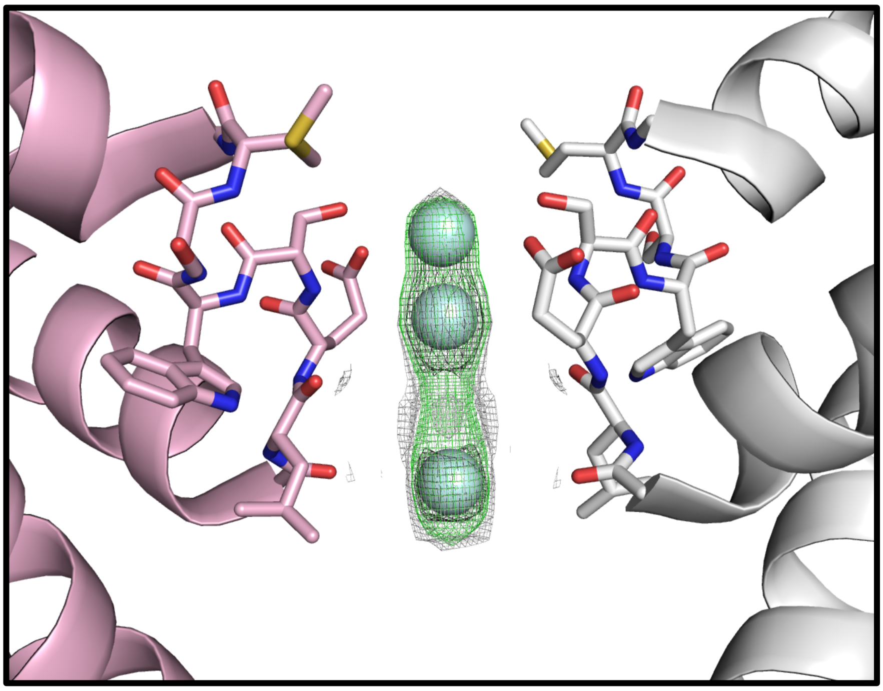

Appendix Figure S6: Refinement of sodium ions and water molecules in the AB and CD tetramers. Structures were refined by the method described for Appendix Figure S1A and are deposited as PDBID 5BZB. A) Occupancies and B-factors. The actual occupancies of the ions indicated by * are 1.0 as a result of being located on a symmetry axis. B) Locations and identities of ions and waters referred to in the above table with respect to the structures.

A)

Atom	B-factor (Å²)	Occupancy
	AB tetramer	
Sodium		
401	67.4	0.5(1.0)*
402	59.0	0.5(1.0)*
403	62.2	0.5(1.0)*
Water		
501	44.3	0.5
502	40.1	0.5
503	21.6	0.5
504	96.9	0.5
505	86.6	0.5
506	37.5	0.5
507	55.0	0.5
	CD tetramer	
Sodium		
401	70.6	0.5(1.0)*
402	23.5	0.5(1.0)*
403	47.3	0.5(1.0)*
Water		
501	41.4	0.5
502	44.7	0.5
503	33.8	0.5
504	67.8	0.5
505	30.0	0.5
506	26.6	0.5
507	20.8	0.5
508	22.6	0.5

B)

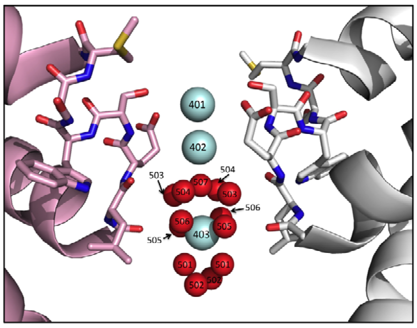

AB tetramer

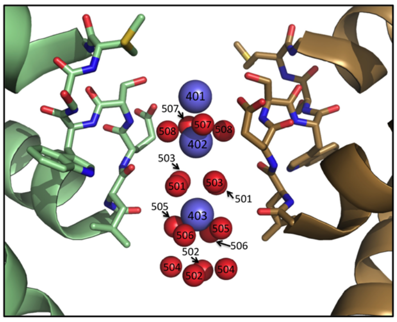

CD tetramer

Appendix Figure S7: Electrostatic potential energy calculations for ions of different radii and charges in wildtype and mutant NavMs pores. The calculation is done by moving a single ion along the direction of the pore. For clarity the relative positions of the ions within the crystal structure are indicated by the cartoon representation behind the plot, with the sites of the ions seen in the AB tetramer indicated as blue/cyan circles.

A) Wildtype (^{177}LESWSM^{182}).

B) E178D mutant (^{177}L<u>D</u>SWSM^{182}).

The mutant sequence is predicted to make the channel more calcium-selective in the SF than the wildtype pore, but still be inhibitory to ion translocation due to unfavourable interactions with the region near the activation gate at the intracellular surface of the pore, a result consistent with the permeability measurements.

A

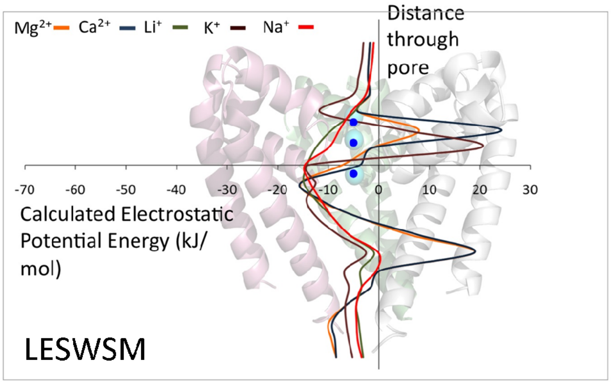

B

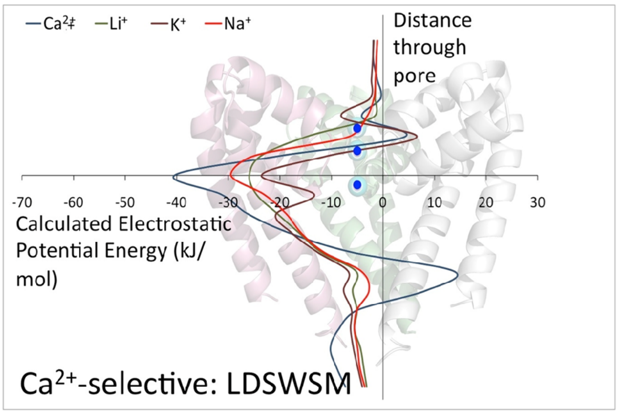

Appendix Figure S8: The E178D mutant single channel conductance.
A) Sample single-channel events recorded from an inside-out patch. Channel-opening events were triggered by variable depolarizations from a holding potential of -170 mV in control conditions (blue traces) and after exchange of the intracellular side (bath) of the patch compartment with 500 µM QX-314 (red traces; see methods).
B) The resulting event amplitudes are plotted as a function of voltage, and the conductance was estimated by fitting the data to a linear relationship (n = 3 cells, Error = S.D.).

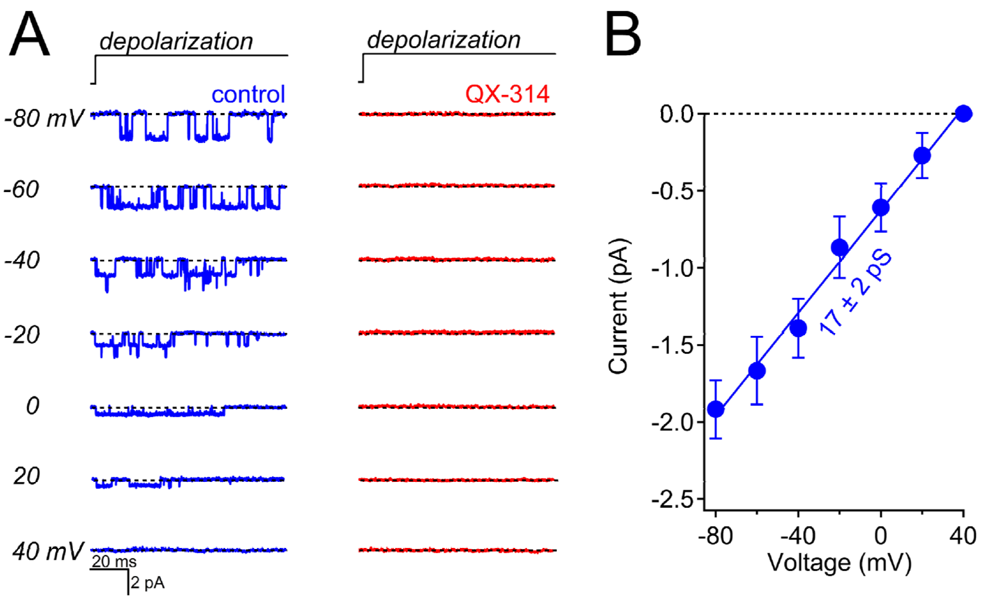

Appendix Figure S9: Superposition of the sodium ions in the NavMs and the calcium ions in the CavAb (PDBID: 4MS2) selectivity filters.

A) The selectivity filter residue side chains from NavMs and CavAb were superposed with LSQKAB using residues 142-152 and 195-204 (NavMs numbering) from each of the four monomers. The NavMs AB tetramer is shown in white and light pink (as in Figure 2A) and the CavAb tetramer is shown in green. The NavMs sodium ions are shown as cyan spheres, and the CavAb calcium ions are shown as green spheres.

B) Electrostatic potential energy calculations for ions of different radii and charges passing through the CavAb selectivity filter sequence ^{177}LDDWAD^{182}. It can be seen that calcium ions are highly favoured by this SF sequence.

A

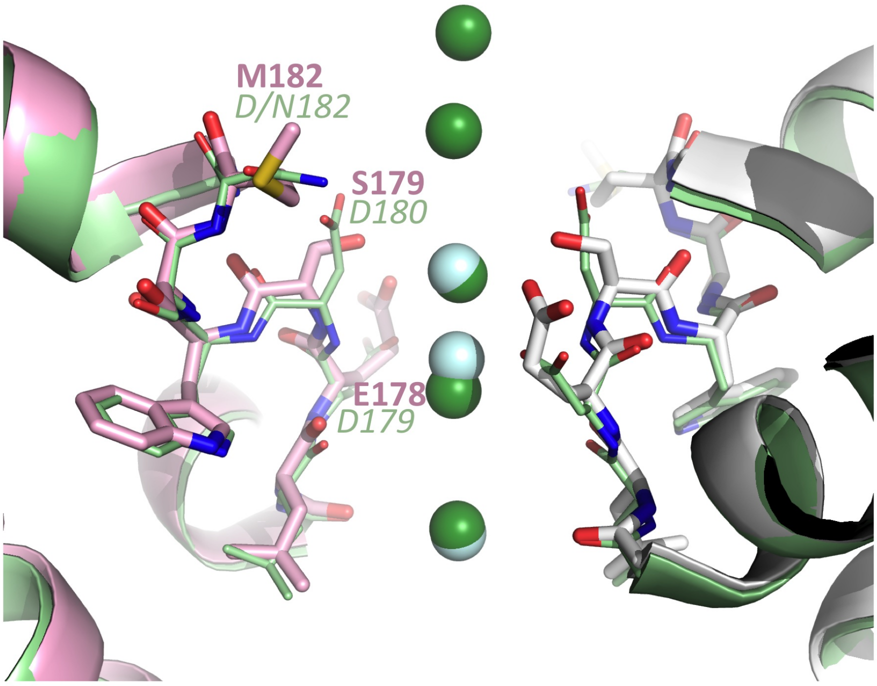

B

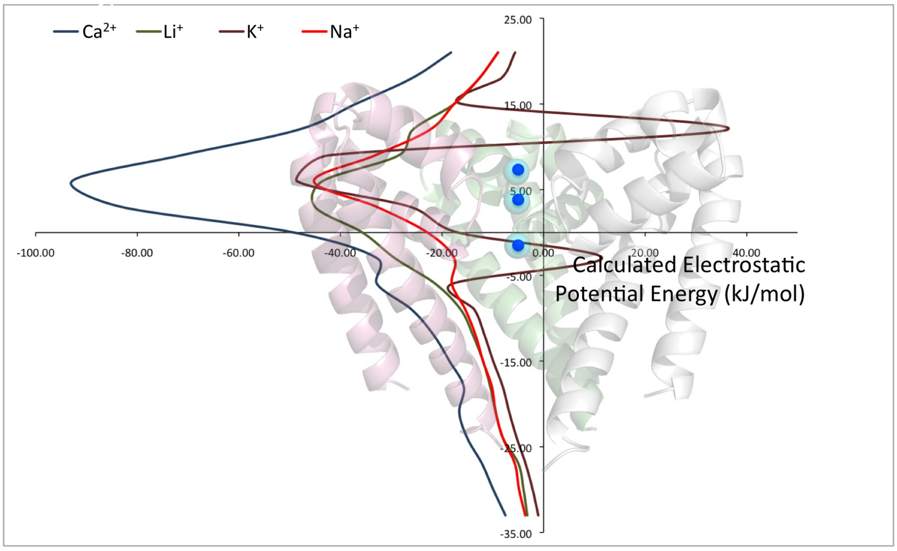

Appendix Figure S10: Lack of NavMs sodium current antagonism by silver and cadmium ions.

A, B) Sodium current (I_{Na}) recorded from HEK-293T cells transfected with NavMs were voltage clamped in the whole-cell configuration. *Left:* I_{Na} traces activated by a 0.2 Hz train of 0.3 s depolarizations to -30 from -180 mV. *Right:* The onset of block was assessed by subtracting the control I_{Na} rundown regression (red line) from I_{Na} after 1.5 min of extracellular application of transition metals at the concentrations indicted by the colored boxes (see methods for details). The black boxes are the control application of 0.3% DMSO at the maximum concentration used as a vehicle for the divalent metals. (± SEM, n = 4-5 cells).

C) Lack of a concentration-I_{Na} block relationship (± SEM, n = 4-5 cells) for both Cd^{2+} and Ag^{+}. The effects of Ag^{+} on I_{Na} were limited to 200 µM due to the solubility limitation of silver in the recording saline.

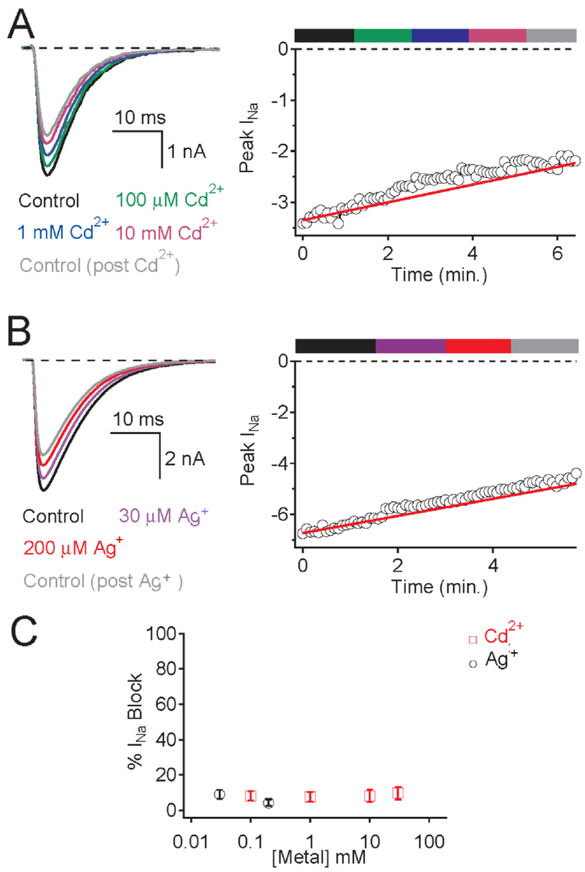

Appendix Table:

Appendix Table S1: Data Collection and Refinement Statistics (Molecular Replacement) for Wildtype and Mutant NavMs Pore Crystals

**Ion or Mutant**	**Wildtype**	**E178D**
**PDB Code**	**5BZB**	**4X88**
**Data Collection**		
Number of crystals	1	1
Space group	$C222_1$	$C222_1$
Cell dimensions		
a, b, c (Å)	80.15,334.38, 80.21	80.23,336.02, 80.30
α, β, γ (°)	90.0, 90.0, 90.0	90.0, 90.0, 90.0
Resolution (Å)	57.8-2.7(2.8-2.7)*	50.0-3.5(3.8-3.5)
R_{merge}	0.245 (0.844)	0.274 (0.594)
R_{pim}	0.07 (0.268)	0.146 (0.315)
$CC_{1/2}$ (%)	99.9 (85.3)	99.6 (95.4)
I/σI	9.5 (3.2)	7.7 (3.9)
Completeness (%)	99.9 (99.9)	99.9 (99.9)
Redundancy	12.3 (10.7)	6.5 (6.5)
**Refinement**		
Resolution (Å)	45.8-2.7 (2.8-2.7)	45.9-3.5 (3.8-3.5)
No. reflections	30134 (2901)	14163 (2856)
R_{work} / R_{free}	17.2/21.2 (18.4/23.3)	19.8/20.5 (18.9-21.0)
No. atoms		
Protein	2856	2852
Ligand/ion	158	127
Water	362	6
B-factors		
Protein	45.8	34.7
Ligand/ion	59.5	23.7
Water	51.3	30.0
R.m.s.deviations		
Bond lengths (Å)	0.016	0.014
Bond angles (°)	1.89	1.81
Ramachandran Angles		
Favoured (%)	96.1	95.2
Outliers (%)	0.0	0.0

*Values in parentheses are for the highest resolution shell
